# Reduction of Diphenyl Diselenide and Analogs by Mammalian Thioredoxin Reductase Is Independent of Their Gluthathione Peroxidase-Like Activity: A Possible Novel Pathway for Their Antioxidant Activity

**DOI:** 10.3390/molecules15117699

**Published:** 2010-10-28

**Authors:** Andressa Sausen de Freitas, Alessandro de Souza Prestes, Caroline Wagner, Jéssie Haigert Sudati, Diego Alves, Lisiane Oliveira Porciúncula, Ige Joseph Kade, João Batista Teixeira Rocha

**Affiliations:** 1 Departamento de Química, Centro de Ciências Naturais e Exatas, Universidade Federal de Santa Maria, 97105-900, Santa Maria RS, Brazil; E-Mails: andressasausen@yahoo.com.br(A.S.F.); prestes_asp@hotmail.com(A.S.P.); carolwagner@ibest.com.br(C.W.); jhsudati@gmail.com(J.H.S.); 2 Departamento de Química, Universidade Federal de Pelotas, Brazil; E-Mail: dalves@gamail.com (D.A.); 3 Departamento de Bioquímica, Instituto de Ciências Básicas da Saúde, Universidade Federal do Rio Grande do Sul, 90035-003, Porto Alegre, RS, Brazil; E-Mail: loporciuncula@yahoo.com.br (L.O.P.); 4 Departament of Biochemistry, Federal University of Technology of Akure, Akure, Ondo, Nigeria; E-Mail: ijkade@yahoo.com (I.J.K.)

**Keywords:** glutathione peroxidase, thioredoxin reductase, organoselenium compounds

## Abstract

Since the successful use of the organoselenium drug ebselen in clinical trials for the treatment of neuropathological conditions associated with oxidative stress, there have been concerted efforts geared towards understanding the precise mechanism of action of ebselen and other organoselenium compounds, especially the diorganyl diselenides such as diphenyl diselenide, and its analogs. Although the mechanism of action of ebselen and other organoselenium compounds has been shown to be related to their ability to generally mimic native glutathione peroxidase (GPx), only ebselen however has been shown to serve as a substrate for the mammalian thioredoxin reductase (TrxR), demonstrating another component of its pharmacological mechanisms. In fact, there is a dearth of information on the ability of other organoselenium compounds, especially diphenyl diselenide and its analogs, to serve as substrates for the mammalian enzyme thioredoxin reductase. Interestingly, diphenyl diselenide shares several antioxidant and neuroprotective properties with ebselen. Hence in the present study, we tested the hypothesis that diphenyl diselenide and some of its analogs (4,4’-bistrifluoromethyldiphenyl diselenide, 4,4’-bismethoxy-diphenyl diselenide, 4.4’-biscarboxydiphenyl diselenide, 4,4’-bischlorodiphenyl diselenide, 2,4,6,2’,4’,6’-hexamethyldiphenyl diselenide) could also be substrates for rat hepatic TrxR. Here we show for the first time that diselenides are good substrates for mammalian TrxR, but not necessarily good mimetics of GPx, and *vice versa*. For instance, bis-methoxydiphenyl diselenide had no GPx activity, whereas it was a good substrate for reduction by TrxR. Our experimental observations indicate a possible dissociation between the two pathways for peroxide degradation (either via substrate for TrxR or as a mimic of GPx). Consequently, the antioxidant activity of diphenyl diselenide and analogs can be attributed to their capacity to be substrates for mammalian TrxR and we therefore conclude that subtle changes in the aryl moiety of diselenides can be used as tool for dissociation of GPx or TrxR pathways as mechanism triggering their antioxidant activities.

## 1. Introduction

In the last three decades, the concept that selenium-containing molecules may be better nucleophiles than classical antioxidants has led to the design of synthetic organoselenium compounds such as ebselen, (2-phenyl-1,2-benzisoselenazol-3(2*H*)-one) that was reported to exhibit borderline success in clinical trials, especially in pathologies associated with oxidative stress [1,2,3]. These promising results have stimulated the interest in the area of organochalcogen synthesis with particular emphasis on their biological properties [4,5,6,7]. 

The antioxidant properties of ebselen and other organochalcogenides have been linked mainly to glutathione peroxidase- or thiol-peroxidase-like activities (*i.e.*, these compounds can decompose peroxides using either reduced glutathione or other thiols) [4,5,8,9]. However, the Holmgren group recently demonstrated that ebselen is also a substrate for mammalian Trx reductase (TrxR) and can be reduced by electrons derived from NADPH, forming its selenol/selenolate intermediate [10,11]. These authors have also observed that the selenol/selenolate of ebselen can be oxidized to ebselen diselenide, which is also a good substrate for mammalian Trx reductase [10]. Consequently, ebselen and its diselenide can be reduced to a common selenol/selenolate that can efficiently decompose hydrogen peroxide. Most importantly, the Holmgren group elegantly obtained persuasive experimental points of evidence indicating that the decomposition of hydrogen peroxide by ebselen via Trx reductase was more efficient than that the thiol-peroxidase-like activity [10].

Mammalian TrxRs are large selenoproteins with structures showing a close homology to glutathione reductase, but with an elongation containing a unique catalytically active selenolthiol/selenenylsulfide in the conserved C-terminal sequence Gly-Cys-Sec-Gly. It also has a remarkable wide substrate specificity, reducing not only different thioredoxins but also selenite, selenodiglutathione, selenocystine, ebselen and its diselenide [10,12,13,14,15]. During its catalytic cycle, the selenocysteinyl residue of TrxR interacts with a cysteinyl residue, forming an enzyme intermediate that transitorily possesses a -S-Se- bond. This bond is subsequently reduced to -SH and SeH, which participate in the reduction of the disulfide from Trx or disulfides and diselenides from artificial substrates (including ebselen diselenide). 

On the other hand, classical GPx (GPx1) is a selenium-containing antioxidant enzyme composed of four identical subunits, and each subunit contains one selenocysteine residue [16,17]. This enzyme reduces hydrogen peroxide and a broad scope of organic hydroperoxides using GSH as electron donor. However, it does not reduce the hydroperoxy groups of complex lipids, a reaction mediated by a phospholipid hydroperoxide glutathione peroxidase (GPx4) [16]. In contrast to TrxR, the catalytic cycle of Seleno-GPx enzymes involves the oxidation of the selenol to selenenic acid that is subsequently reduced back to selenol in two steps by the consumption of an equivalent of two molecules of GSH. Consequently, the formation of -S-Se- bond involves first the interaction of the oxidized selenium atom from the selenenic acid in the catalytic centre of the enzyme with the low molecular weight GSH. 

From a mechanistic point of view, the interaction of a thiol with a diselenide can form the corresponding disulfide and selenol intermediates [5,18]. The selenol/selenolate can subsequently be oxidized to the selenenic acid by hydrogen peroxide [10,18], which can be a low-molecular weight analog of the selenocysteinyl residue from the active centre of GPx. In the presence of excess of thiol, the selenenic acid can be reduced back to selenol that hypothetically could maintain a mimetic catalytic cycle of GPx (glutathione peroxidase- or thiol-peroxidase like activity, depending on the reducing thiol considered) [5,8]. Alternatively, in the absence of an excess of a low molecular weight reducing thiol, TrxR can reduce ebselen and/or ebselen diselenide using reducing equivalents from NADPH [10,11].

**Figure 1:**
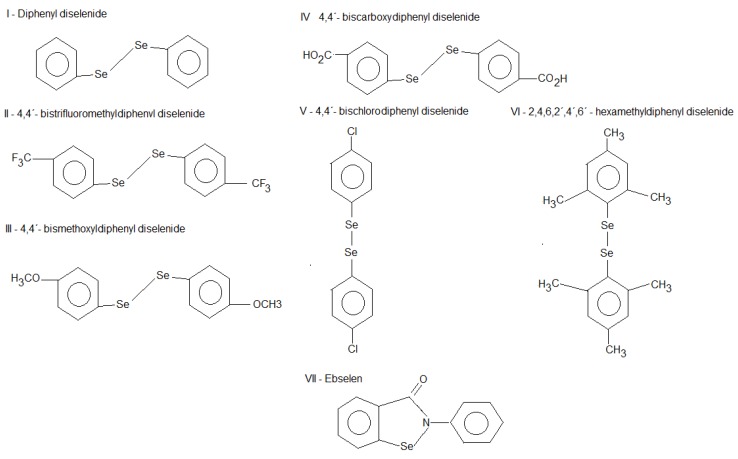
Structures of diselenide compounds.

Diphenyl diselenide and analogs share several antioxidant and neuroprotective properties with ebselen [5,19,20,21,22,23,24,25,26] and they are also mimetics of native GPx enzyme [5,8]. However, there is a dearth of information in the literature indicating that simple diorganyl diselenides such as diphenyl diselenide and its analogs can be substrates for TrxR. Hence, taking into account that the TrxR can be the major pathway for the observed reduction of ebselen or its diselenide form *in vivo*, and also that simple aryl diselenide compounds are analogs of ebselen diselenide, we tested the hypothesis that these compounds (Figure 1) could also be substrates for TrxR. 

## 2. Results

### 2.1. Thiol Oxidase Activity of Selenium Compounds

Table 1 shows the thiol oxidase activity of selenium compounds. Apparently, diphenyl diselenide exhibited a more profound thiol oxidase activity when compared to other organoselenium tested. In fact, diphenyl diselenide caused an almost complete oxidation of DTT after 60 min of reaction. The ability of the seven organoselenium tested to oxidize thiol is in the order: diphenyl diselenide > bischlorodiphenyl diselenide > bisfluoromethyldiselenide > hexamethyldiphenyl diselenide ≈ ebselen > bismethoxydiphenyl diselenide > biscarboxydiphenyl diselenide (p < 0.01, one-way ANOVA followed by Duncan’s test). It is noteworthy that DTT oxidation determined in the presence of hexamethyl-diphenyl diselenide or ebselen was not different from that determined in the absence of chalcogens (control). In addition, bismethoxydiphenyl diselenide and biscarboxydiphenyl diselenide did not exhibit significant thiol oxidase activity. Indeed, they decreased DTT oxidation. 

### 2.2. Ebselen and Diphenyl Diselenide Compounds Are Substrates for Hepatic Mammalian Thioredoxin Reductase (TrxR) 

**Figure 2:**
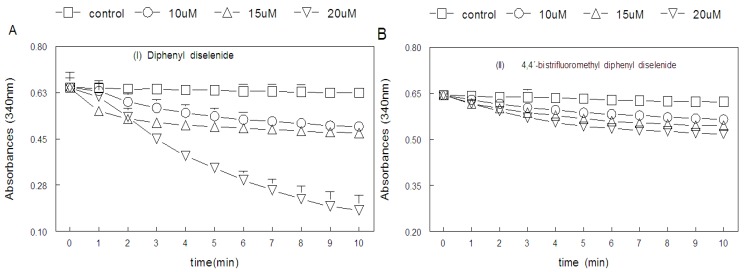

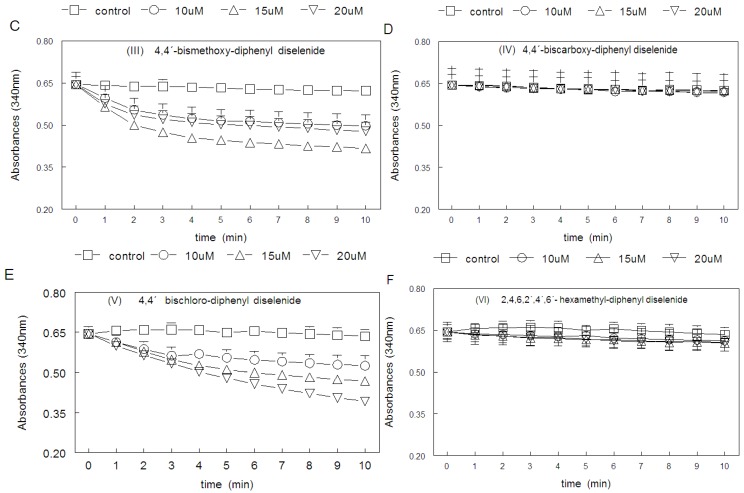
Reduction of diselenide compounds I (A), II ( B), III (C), IV (D), V (E) e VI (F) by NADPH catalyzed by mammalian Thioredoxin Reductase (TrxR). Enzyme was mixed with a medium containing 50 mM Tris-HCl, 1 mM EDTA, pH 7,5 and then reaction was started by adding NADPH (final concentration 100 µM). 0 (**□**), 10(O), 15(∆), 20(∇) µM diselenide compounds. Statistical analysis were performed by three-way ANOVA (six diselenides *×* four concentrations *×* 11 sampling points). Data analysis yielded a significant diselenide *×* concentration *×* time interaction F(150, 1,440) = 42.5; p < 0.000001, which indicates that the consumption of NADPH was dependent on the concentration, on the type of compound and on the sampling time.

**Figure 3:**
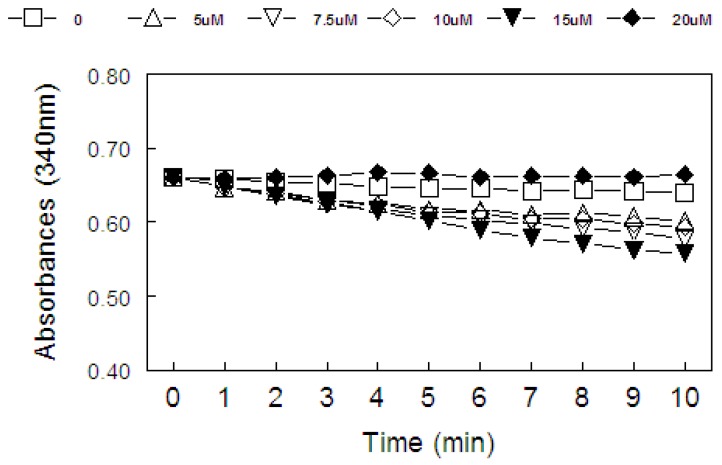
Reduction of ebselen (0, 5, 7.5, 10, 15 or 20 µM) by NADPH catalyzed by mammalian TrxR. Enzyme was mixed with a medium containing 50 mM Tris-HCl, 1 mM EDTA, pH 7.5 and, then reaction was started by adding NADPH (final concentration 100 µM). Statistical analysis were performed by two-way ANOVA (six concentrations × 11 time points). Data analysis yielded a significant concentration *×* time interaction [F(50, 360) = 24.6; p < 0.000001], which indicates that the consumption of NADPH determined in the presence of ebselen depended both on its concentration and on time of sampling.

Figure 2 and Figure 3 show the effect of organoselenium compounds on the oxidation of NADPH in the presence of partially purified hepatic mammalian TrxR. Diphenyl diselenide, bisfluromethyldiphenyl diselenide, bismethoxydiphenyl diselenide and bischlorodiphenyl diselenide (at concentrations of 10, 15 and 20 µM) and ebselen (at concentrations varying from 5 to 15 μM) stimulated NADPH oxidation in the presence of partially purified hepatic mammalian Trx Reductase (TrxR), indicating that they are substrates for hepatic mammalian TrxR. In fact, diphenyl diselenide was a better substrate than its analogs particularly when tested at 20 μM (Figure 2, three-way ANOVA, see Figure legend for details) and ebselen (Figure 3, separate analysis performed only for diphenyl diselenide and ebselen using 5-15 μM by two-way ANOVA, data not shown). Oxidation of NADPH observed in the presence of these selenium compounds were almost completely blocked by 1 μM AuCl3 (more than 90%), Furthermore, we confirmed the formation of reduced intermediates of diselenides that were substrate for TrxR by reacting aliquots of the enzymatic reaction of NADPH oxidation with DTNB (here we have included 5 μM of AuCl_3_ to inhibit any further reduction of DTNB by NADPH catalyzed by TrxR during color development (data not shown). 

### 2.3. Thiol Peroxidase-Like Activity of Diphenyl Diselenide and its Analogs

**Figure 4:**
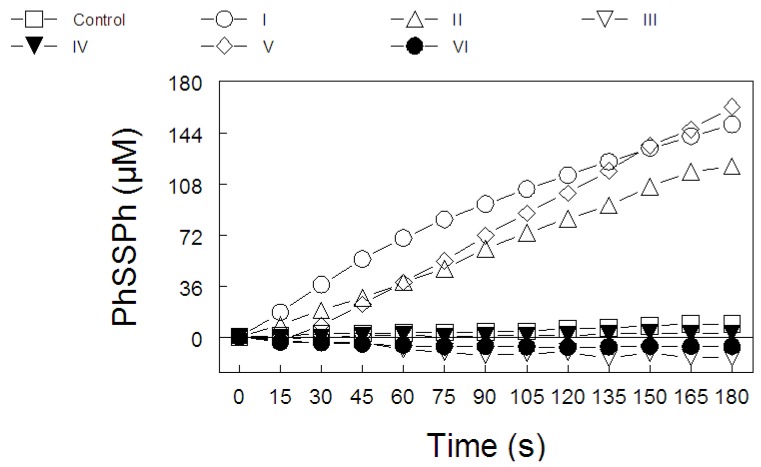
GPx like behavior of diselenide compounds: (**I**) diphenyl diselenide, (**II**) bistrifluoromethyldiphenyl diselenide, (**III**) bismethoxydiphenyl diselenide, (**IV**) biscarboxydiphenyl diselenide, (**V**) bischlorodiphenyl diselenide and (**VI**) hexamethyl-diphenyl diselenide. Two hundred µM of diselenide compounds was added. Statistical analysis were performed by two-way ANOVA (seven compounds *×* 13 sampling times). Data analysis yielded a significant compound type *×* time of sampling interaction [F(72, 504) = 57.9 p < 0.01), which indicates that PhSSPh production varied as a function of sampling time and the diselenide considered.

**Figure 5:**
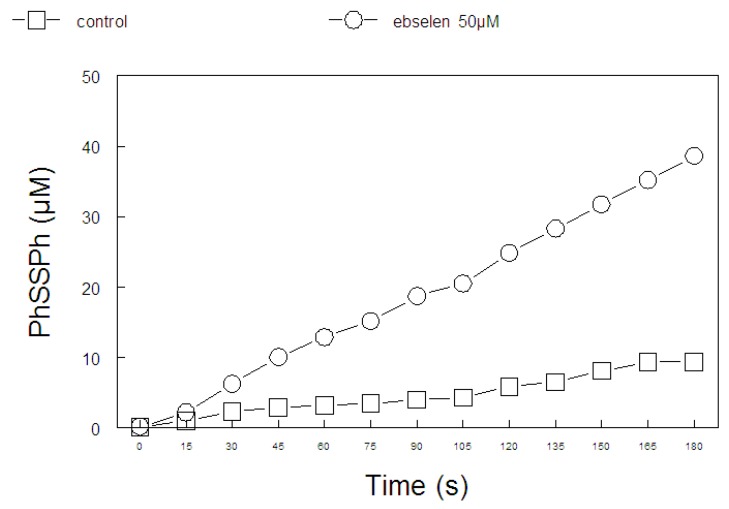
GPx like behavior of ebselen. Fifty µM of ebselen was tested. Statistical analysis were performed by two-way ANOVA (two concentrations *×* 13 times of sampling). Data analysis yielded a significant concentration *×* sampling time interaction [F(12,144) = 228,4; p < 0.000001], which indicates that ebselen caused an increase in PhSSPh formation that was time dependent.

The results obtained from the analysis of the thiol peroxidase-like activity of 200 μM of diphenyl diselenide and its analogs and ebselen (50 μM) are presented in Figure 4 and Figure 5, respectively. In Figure 4, we observe that bischlorodiphenyl diselenide and bistrifluoromethyldiphenyl diselenide decomposed hydrogen peroxide with a similar efficiency, when compared to diphenyl diselenide. In contrast, bismethoxydiphenyl diselenide, biscarboxydiphenyl diselenide and hexamethyldiphenyl diselenide had no thiol-peroxidase-like activity. In Figure 5, the thiol peroxidase-like activity of ebselen could not be measured at 200 μM, because the readings at 305 nm were higher than the limit of the spectrophotometer. 

### 2.4. Correlation between Thiol Oxidase, Thiol Peroxidase-Like and Effectiveness of Diphenyl Diselenide, its Analogs and Ebselen to be Substrate for TrxR

**Figure 6:**
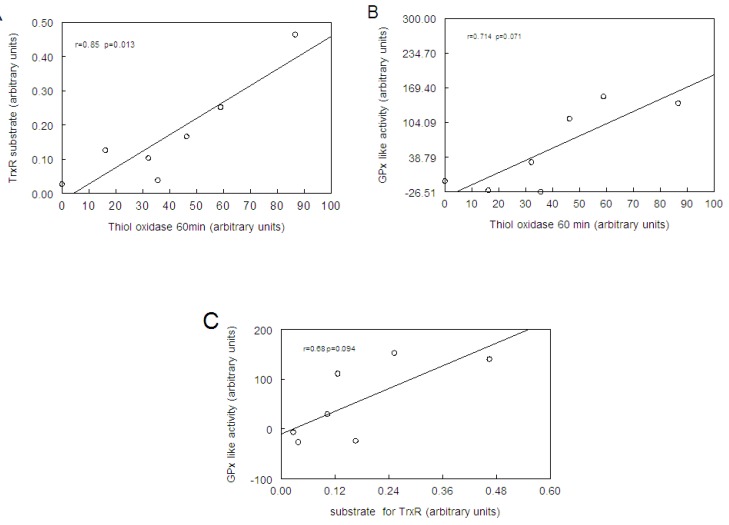
Correlation analysis (by Sperman rank test) between the thiol oxidase activity with the effectiveness of diselenides and ebselen as substrate of hepatic mammalian TrxR (A) or with thiol peroxidase-like activity (B) or and between thiol-peroxidase like activity and the effectiveness of diselenides and ebselen as substrates for TrxR (C).

Figure 6 show the correlation analysis (Spearman rank test) between the thiol oxidase activity, thiol peroxidase-like activity, and the effectiveness of diselenides and ebselen to be substrates for hepatic mammalian TrxR. Figure 6a shows a significant correlation between thiol oxidase and the effectiveness of diselenides and ebselen to act as substrates of TrxR (r = 0.85; p = 0.013). In addition, Figure 6b shows a tendency for a significant correlation between thiol oxidase activity with thiol-peroxidase-like activity (r = 0.71, p = 0.07). However, as shown in Figure 6c, statistical analysis revealed no significant correlation between thiol-peroxidase like activity and the effectiveness of diselenides and ebselen to act as substrates for TrxR (r = 0.67, p = 0.094). From a qualitative point of view, it can easily be observed that bismethoxydiphenyl diselenide had a high activity as substrate for mammalian TrxR (Figure 2C), whereas it has no activity as a mimetic of GPx (Figure 4). Similarly, bistrifluoromethyldiphenyl diselenide had a weak activity as substrate for TrxR (Figure 2B), whereas it has a high thiol-peroxidase-like activity (Figure 4). The relative activities of all three determinations in relation to control groups are presented in Table 2. 

## 3. Discussion 

Organoseleno compounds, particularly, 2-phenyl-1,2-benzisoselenazol-3(2H)-one or ebselen were used in clinical trials about 10 years ago for the treatment of neuropathological conditions associated with oxidative stress [1,2,3]. Based on literature data, these authors attributed the neuroprotective effects of ebselen to its anti-inflammatory properties and its thiol peroxidase-like activity (for reviews about the antioxidant properties of ebselen see [4,5]). In fact, the concept that ebselen (and other organocalchogens) could be used for the treatment of human diseases associated with oxidative stress was linked essentially to their GPx-like activity. Based on this assumption, we have investigated the pharmacological and antioxidant properties of diphenyl diselenide, the simplest of the diaryl diselenide compounds, in different *in vitro* and *in vivo* models of oxidative stress. In fact, literature demonstrates a higher GPx-like activity for diphenyl diselenide (about two times), than ebselen [8,27]. Furthermore, diphenyl diselenide and analogs also have higher anti-inflammatory activity than ebselen [28]. Although the GPx-like activity of organoseleno compounds could account for their pharmacological properties, there is no study showing a clear correlation between the thiol-peroxidase like activity and *in vivo* protective effects in rodents. Some of diselenides used here (diphenyl diselenide, p-Cl-, F_3_C-) have antioxidant activity (determined by TBARS) in the µM range, when brain was used as the source of lipids. However, the compound with highest GPx-like activity (p-Cl-) was the one with a weak antioxidant activity. These results indicate that the antioxidant activity (as determined by the production of TBARS) is also not directly related to GPx-like activity.

More recently, the Holmgren group have elegantly demonstrated that ebselen and its diselenide were good substrates for human thioredoxin reductase [10,11]. Furthermore, they have also clearly demonstrated that the antioxidant activity of ebselen and its diselenide is related to their reductions by mammalian thioredoxin reductase (TrxR) and thioredoxin (Trx) producing ebselen selenol and that the TrxR pathway could more efficiently decompose hydrogen peroxide than the GPx-like pathway [10]. Consequently, the potential participation of simple diaryl diselenides as substrates for TrxR could explain their antioxidant and pharmacological properties. 

In the present study, we observed that the thiol oxidase-like ability (ability to oxidize thiols in the absence of peroxide), GPx-like activity (oxidation of thiols determined in the presence of peroxide) and the ability of these compounds to be reduced by TrxR varied greatly depending on the substitution in the aromatic moiety. Diphenyl diselenide, bisfluoromethyl diselenide and bischlorodiphenyl diselenide exhibited all these three activities. In contrast, bismethoxydiphenyl diselenide has a high activity as a substrate for mammalian TrxR, whereas it has no GPx mimetic activity. In the case of bistrifluoromethyldiphenyl diselenide, on the other hand, we have to emphasize that it exhibited a weak activity as a substrate for TrxR (Figure 2B), whereas it has a high thiol-peroxidase-like activity. Biscarboxydiphenyl diselenide and hexamethyldiphenyl diselenide did not exhibit any of these activities in appreciable amounts. Although the thiol oxidase activities of a diselenide can indicate a potential pro-oxidant property and, consequently, can give some indication about its potential toxicity [5,29], its interaction with thiols is critical for forming the selenol/selenolate for degradation of peroxides. In the present study, it is obvious that diselenides that exhibit appreciable thiol peroxidase like activity and/or that were good substrates for TrxR displayed thiol oxidase activity, except bismethoxydiphenyl diselenide. In fact, there is a positive correlation between thiol oxidase activity and the capacity of these compounds to be reduced by TrxR. In addition, the statistical analysis of the GPx of the organoselenium compounds showed a tendency towards a positive correlation between thiol oxidase and GPx-like activity. However, there is no significant correlation between thiol peroxidase-like activity and the capacity of diselenides to be reduced by TrxR indicating that for some compounds there is no relationship between their thiol peroxidase like activity and their ability to be a suitable substrate for TrxR. For instance, as indicated in Table 2, bismethoxydiphenyl diselenide was a good substrate for mammalian TrxR but has neither thiol peroxidase-like activity nor thiol oxidase activity. In fact, it exhibits thiol oxidase activity lower than the control. The apparent paradoxical effects of biscarboxy- and bismethoxydiphenyl diselenide on DTT oxidation are not easily explainable. Particularly if one consider that the carboxy group is an electron withdrawing group (which is expected to stabilize the selenol and, consequently, to diminish its catalytic properties as thiol oxidizing agent); and the methoxy- group is an electron donating group and should increase the reactivity of the selenol/selenolate formed. For the case of the biscarboxy-substituent, the stability of the selenol/selenolate might permit it to reduce back the oxidized DTT to DTT before being oxidized by molecular oxygen (see [5] for details about the catalytic oxidation of thiols by diphenyl diselenide), which could explain its “protective” effect on thiol oxidation. However, this explanation cannot be applied to bismethoxydiphenyl diselenide. One factor that could contribute to these differences is the presence of a relatively bulky group with oxygen in both R groups. Thus, the presence oxygen in these groups and steric hindrance could difficult the interaction of these two compounds with DTT.

The diselenides used here are structurally similar, thus it is difficult to explain why they exhibit differential activities as either thiol oxidase or thiol peroxidase mimic and/or their capacity to be reduced by TrxR. Previously, we have observed that a bulky organic moiety markedly decreased the antioxidant and thiol-peroxidase-like activity of a diselenide analog of cholesterol [30]. Here, the organic moiety substitutions in the diselenides examined in the present study were relatively smaller molecules when compared to cholesterol, but the present data showed a marked decline in their reactivity as thiol oxidase, thiol peroxidase mimic and their ability to act as substrates for TrxR. For instance, biscarboxydiphenyl diselenide and hexamethyldiselenide were almost inactive either as GPx mimic or as substrates for TrxR. Furthermore, we also observe that the GPx mimetic ability of the diselenides could be altered due to electronic effects. For example, electron donating groups (in the case of hexamethyl and bismethoxydiphenyl deselenide) caused a complete loss of thiol peroxidase activity. On the other hand, introduction of electron withdrawing groups (chloro- or triflouromethyl-) was generally associated with non-significant changes in thiol peroxidase-like activity (the exception was the introduction of a carboxyl group that caused a complete inactivation of all the activities). It is apparent however, that electronic effect may not be a critical factor that could determine the capacity of the diselenides to be reduced by TrxR. In fact, the introduction of an electron donating group could either be associated with a high (bismethoxydiselenide) or low (hexamethyl-) capacity to be reduced by TrxR. In contrast to GPx-like activity, introduction of electron withdrawing groups (chloro or trifluoromethyl) were associated with high and moderate capacity to be substrate for TrxR (Table 2). Consequently, we can suppose that the ability of the diselenides to be substrates for TrxR may be related to steric effect which could be a more important factor than factors that could interfere with the selenol/selenolate stability such as electronic effect.

The results presented here indicate that diphenyl diselenide and some of its analogs were good substrates for mammalian TrxR, which indicate that theirs *in vitro* and *in vivo* antioxidant properties could also be associated with their interaction with the thioredoxin-thioredoxin reductase system. These results expand those of Holmgren and collaborators and demonstrated that simple diselenides could also be substrates for TrxR. One aspect that deserves further investigation is whether the thiol oxidase, thiol peroxidase and the capacity to be reduced by TrxR could be used to predict the potential toxicity and/or pharmacology of these molecules. In fact, considering the thiol oxidase activity of diselenides and, from a linear point of view, one should expect a higher toxicity for diphenyl diselenide than their analogs. However, among the diselenides tested in the present study, diphenyl diselenide had a good thiol peroxidase-like activity and a moderate capacity to be reduced by TrxR. 

## 4. Experimental 

### 4.1. Materials and Enzyme

All chemicals were of analytical grade and were obtained from either Sigma Aldrich or Fluka. TrxR from rat liver was purified essentially as described by Hill *et al.* [31].

### 4.2. Thioredoxin Reductase Assay

TrxR activity was determined according to Zhao and Holmgren [10]. Activity was performed in a buffer containing 50 mM Tris-HCl, 1 mM EDTA, pH 7.5, 100 μL of TrxR (10 μg protein/mL of reaction medium) and 100 μM of NADPH. The volume of enzyme was chosen based on previous experiments (data not shown), in which trials were conducted with concentrations of 2.5, 5.0, 7.5, 10, 12.5, 15.0 and 20 μg of the partially purified protein/mL of reaction medium and we have observed a linear reaction from 5.0 to 12.5 μg/mL when diphenyl diselenide and 4,4’-bischlorodiphenyl diselenide were used as substrate (data not shown). Enzyme reaction was started with the addition of different amount of organoselenium compounds. 

### 4.3. Determination of GPx like Activity

Catalytic GPx model reaction (H_2_O_2_ + 2PhSH→2 H_2_O + PhSSPh) [32] was initiated by the addition of H_2_O_2_ (10.4 mM) to a solution of PhSH (5 mM) in presence of diselenide compounds (**I**-**VII**), separately, and was monitored by UV spectroscopy at 305 nm (3 min), at least more than three times under the same conditions.

### 4.4. Thiol Oxidase Activity

Thiol oxidase activity was determined according to the Ellman method [33]. Diselenide compounds (100 µM) were dissolved in DMSO and they were incubated (37 °C) separately in a medium reaction containing 50 mM Tris/HCl buffer (pH 7.4) and the sulphydryl compound dithiothreitol (DTT, 0.5 mM) in a final volume of 1,800 µL. An aliquot (100 µL) from each sample was removed at different times during the reaction (0, 30, 60 and 120 mins) and mixed in a system containing 25 µL of 5,5’ dithiobis -2-nitrobenzoic acid (DTNB) 10 mM and 100 mM Tris/HCl buffer (pH 7.4) in a final volume of 1800 µL. Samples were read at 412 nm. DTT was used as the standard. 

### 4.5. Statistical Analysis

Statistical analysis were performed using one-way (thiol oxidation), two-way (thiol peroxidase activity) and three-way (TrxR determinations) ANOVA. Univariate analysis followed by Duncan´s multiple range test were performed where appropriate. Correlation between thiol oxidase activity, thiol peroxidase-like activity and effectiveness of diphenyl diselenides and ebselen to be substrate for TrxR were analyzed using Spearman rank test. Correlation R values were calculated using the maximal percentage of thiol oxidation in relation to time zero of sampling (*i.e.*, %-SH oxidation of Table 1) for thiol oxidase activity. For TrxR, we have used the activity obtained with 20 µM of diphenyl diselenides in 10 min of reaction. For ebselen, we have used 15 µM because at 20 µM there was a marked inhibition of TrxR. For GPx, we have used the activity obtained with 200 µM of diphenyl diselenides. For ebselen we have used the activity obtained with 50 µM, because it was not possible to determine the activity in the presence of 100 µM of ebselen.

## 5. Conclusions

Thus, the therapeutic potential or toxicity of diselenides may depend on a delicate balance among these three activities (substrate for TrxR; GPx like activity and thiol oxidase activity). However, we must emphasize that some of the pharmacological properties found for these compounds are not only based on their antioxidant characteristics, but also in the interesting ability of these molecules to modulate some pathways and neurotransmitter systems. For instance, 4,4’-bismethoxyldiphenyl diselenide has very interesting antinociceptive [34] and neuroprotective actions [35] that are more related to its ability to modulate receptors than for being an antioxidant. In short, we have demonstrated here for the first time that diphenyl diselenide and its analogs can be substrate for mammalian TrxR, which can explain, at least in part, their *in vivo* antioxidant properties. Furthermore, we also demonstrated for the first time that it is possible to dissociate the two pathways for peroxides degradation (*i.e.*, either via substrate for TrxR or via the mimic of the endogenous antioxidant enzyme, GPx) for structurally related diaryl diselenides. 

## Figures and Tables

**Table 1:** Effects of different selenium compounds on DTT oxidation.

	0 min (nmol -SH/mL)	60 min (nmol -SH/mL)	(%) -SH oxidation)
**Control**	43.48 ± 0.24	30.62 ± 0.25^a^	**29.57**
**I—Diphenyl diselenide**		5.82 ± 0.27^e^	**86.61**
**II—Bistrifluoromethyl-**		23.37 ± 0.36^c^	**46.25**
**III—Bismethoxy-**		36.46 ± 0.83^f^	**16.14**
**IV—Biscarboxy-**		47.19 ± 0.32^h^	**0.0**
**V—Bischloro-**		17.88 ± 0.26^d^	**58.87**
**VI—Hexamethyl-**		28.00 ± 0.40^b^	**35.60**
**VII—Ebselen**		29.51 ± 0.27^a,b^	**32.12**

Values represent mean ± s.e. from three independent experiments performed in duplicate. Selenium compounds were tested at 0.1 mM [(**i**) diphenyl diselenide, (**ii**) bistrifluoromethyl-diphenyl diselenide, (**iii**) bismethoxydiphenyl diselenide, (**iv**) biscarboxydiphenyl diselenide, (**v**) bischlorodiphenyl diselenide, (**vi**) hexamethyldiphenyl diselenide and (**vii**) ebselen]. Reaction was started by adding DTT (to a final concentration of 0.5 mM) and then, aliquots (100 μL) were sampled immediately or 60 min after mixing DTT with compounds. Statistical analysis was performed by One-way ANOVA followed by Duncan´s multiple range test. Means that do not share the same superscript letter are different (p < 0.05).

**Table 2:** Comparisons of the relative activities (GPx, TrxR and Thiol oxidase) of organoseleno compounds.

Sample	GPx	TrxR	Thiol oxidase
**Control**	1.00	1.00	1.00
**I—Diphenyl diselenide**	15.95	8.6	2.93
**II—Bistrifluoromethyl-**	12.84	4.95	1.56
**III—Bismethoxy-**	0.00	11.35	0.55
**IV—Biscarboxy-**	0.31	0.90	0.0
**V—Bischloro-**	17.29	8.8	1.99
**VI—Hexamethyl-**	0.00	2.05	1.20
**VII—Ebselen**	4.12	5.15	1.09

Relative activities were calculated in relation to the control (arbitrary value of 1.00), *i.e*., activity determined in the absence of selenium compounds. For GPx the concentration of the diselenide compounds was 200 μM and for ebselen it was 50 μM. For TrxR assay, calculations were made using the concentration of 15 μM for all the tested selenium compounds.
